# Sirolimus excretion in breast milk: a preliminary investigation

**DOI:** 10.3389/fphar.2026.1859156

**Published:** 2026-06-26

**Authors:** Estela Nogueira, Andreia Bento da Silva, Mónica Rebelo, José António Lopes, Maria do Rosário Bronze, Noélia Duarte

**Affiliations:** 1 Nephrology and Renal Transplantation Department, Unidade Local de Saúde Santa Maria, Lisbon, Portugal; 2 Lisbon Academic Medical Center, Faculty of Medicine, Lisbon University, Lisbon, Portugal; 3 Faculty of Pharmacy, Research Institute for Medicines (iMed.ULisboa), Lisbon University, Lisbon, Portugal; 4 Pediatric Cardiology Department, Unidade Local de Saúde Santa Maria, Lisbon, Portugal; 5 IBET, Instituto de Biologia Experimental e Tecnológica, Oeiras, Portugal

**Keywords:** breast milk, breastfeeding, lactation, sirolimus, transplantation, tuberous sclerosis

## Abstract

**Background:**

Sirolimus is a mammalian target of rapamycin inhibitor that has been used in solid organ transplantation and is now increasingly being administrated to treat various manifestations of Tuberous Sclerosis (TSC). Lack of data regarding its safety during breastfeeding has led to its contraindication by pediatric and breastfeeding guidelines.

**Methods:**

Levels of sirolimus were evaluated in breast milk samples collected during 24 h on the 10th and 15th day postpartum, by a lactating TSC patient chronically medicated with 4 mg of sirolimus per day. Controls were healthy lactating women. Serum levels of sirolimus were also evaluated on the TSC patient on the 10th day postpartum (at the beginning of breast milk collection) but not in the newborn, since the patient was advised not to breastfeed. An ultra-high performance liquid chromatography tandem mass spectrometry (UHPLC–MS/MS) method, including solid–liquid extraction (SLE) for sample preparation, was optimized and validated for the quantitation of sirolimus in breast milk.

**Results:**

After optimization, the SLE-UHPLC-MS/MS method allowed the detection of 100 pg sirolimus per mL^-1^ of breast milk. Sirolimus was detected in all breast milk samples from the TSC patient, ranging from 173 to 492 pg. mL^-1^. However, its concentration was more than 10-fold lower (<10%) than in serum (5900 pg mL^-1^ on the 10th day postpartum, 24 h after the last dose and at the beginning of breast milk collection).

**Conclusion:**

Considering the low oral availability (∼15%) of sirolimus and the concentration found in breast milk, sirolimus is expected to achieve serum concentrations in the newborn below minimum therapeutic concentrations. The assessment of drug exposure in neonatal serum was not possible, as breastfeeding was not recommended. Thus, more studies are needed to characterize infant serum sirolimus concentrations and confirm its safety. Nevertheless, this research provides valuable information for breastfeeding counseling and respective guidelines, especially in patients with an increased risk of prematurity, in whom breast milk benefits are even more important.

## 1 Introduction

Sirolimus is a mammalian target of rapamycin (mTOR) inhibitor. It suppresses T-lymphocyte activation and proliferation in response to antigen and cytokine stimulation and inhibits antibody production. Its immunosuppressive properties have been used in solid organ transplantation for two decades (Mahalati and Kahan, 2001) Additionally, by inhibiting the mTOR pathway, sirolimus prevents the proliferation of lymphangioleiomyomatosis cells. Therefore, it is now increasingly used to treat various manifestations of Tuberous Sclerosis (TSC), with proven success (Li et al., 2019).

Sirolimus is characterized by a prolonged elimination half-life of 44–86 h. Following administration, peak concentrations are typically reached within 0.5–3 h. It exhibits a large apparent volume of distribution (approximately 5.6–16.7 L/kg), reflecting extensive tissue binding and sequestration, particularly within red blood cells. The drug undergoes significant first-pass metabolism mediated primarily by CYP3A4 and P-glycoprotein, contributing to its low oral bioavailability and high interindividual variability. Elimination occurs predominantly via biliary excretion (approximately 91%), with minimal renal clearance (Mahalati and Kahan, 2001).

The use of sirolimus during pregnancy and breastfeeding is still debatable due to paucity of data and has been discouraged by pediatric and breastfeeding guidelines (E-lactation, 2025; Meek et al., 2022). Although preclinical animal data reveal a possible association with miscarriage, malformation, low birth weight and preterm delivery, human data on pregnancy are still sparse, and the risk of teratogenicity has not been yet proven (UK Teratology Information Service, 2025). Based on the lack of evidence, sirolimus use in pregnancy remains restricted to complex and individual cases. Additionally, data on the safety of its use during breastfeeding is also unavailable.

To our knowledge, there is no data regarding sirolimus levels in maternal milk to evaluate its safety. Regarding everolimus, another mammalian target of rapamycin and chemical derivative of sirolimus, only two cases were reported, assessing its levels in patients’ blood, amniotic fluid, umbilical blood and colostrum. In the first case, everolimus concentration was measured once at 48 h postpartum and was below the lower limit of detection (<500 pg. mL^-1^) (Fiocchi et al., 2016).

In the second case, everolimus was present in colostrum up to 12 h after drug administration on the second postpartum day, with concentrations ranging from 33 to 66 pg. mL^-1^, and the highest level occurring 4 h after dosing. Based on these values, it was estimated that the newborn exposure was 4.224 ng/kg/day, several times lower than the weight-adjusted maternal dose and significantly below the immunosuppressant levels to which the fetus was exposed. (Kociszewska-Najman et al., 2017). Considering the high molecular weight of sirolimus (914.2 g/mol) and its extensive plasma protein binding, the excretion of the drug into breast milk is expected to be unlikely (Mahalati and Kahan, 2001; E-lactation, 2025; Gomez-Casado et al., 2025). Only trace amounts have been detected in the milk of lactating rats. (Gomez-Casado et al., 2025).

The aim of this research was to evaluate the levels of sirolimus in breast milk collected during 24 h by a lactating TSC patient chronically medicated with sirolimus. Although the primary aim of this work was not to perform a comprehensive evaluation of the pharmacokinetics of sirolimus excretion into breast milk, the ClinPK checklist was followed to enhance the transparency and completeness of the reporting of the results Supplementary Table S1. (Kanji et al., 2015; Dagge et al., 2022).

It is noteworthy that this patient was treated with sirolimus during pregnancy due to the emergence of life-threatening fetal cardiac rhabdomyoma *in utero*. Postartum, a critical unanswered question was whether breastfeeding could be safely undertaken while the mother remained on sirolimus therapy for renal angiomyolipomas, especially in the context of a newborn with severe prenatal manifestations of tuberous sclerosis complex (Dagge et al., 2022).

## 2 Methods

### 2.1 Breast milk samples

This study was submitted and approved by the Committees of Ethics of Centro Académico de Medicina de Lisboa (CAML - No 196/21, 26/05/2022) and was carried out according to the Declaration of Helsinki. The patient and healthy controls that participated in the study signed an informed consent form. Breast milk was collected from three lactating healthy women (all medication-free Caucasian women, with mean age of 38 ± 2 years old and mean weight 60 kg ± 2) and one lactating patient (Caucasian, 23 years old, weight of 57 kg) with tuberous sclerosis, with normal liver and renal function, followed at a tertiary referral high-risk obstetric center (Unidade Local de Saúde Santa Maria, Lisboa - ULSSM). Following collection, breast milk samples were stored at – 20 °C until analysis.

A breast milk sample was initially collected from the patient with TSC for a preliminary analysis. Following this assessment, the patient reported collecting all breast milk produced during 24 h after sirolimus administration on the 10th postpartum day, beginning at 11 a.m., at 3-h intervals, starting immediately after the sirolimus dose and until 11 a.m. the next day. Breast milk was also evaluated on the 15th day postpartum (from 2 a.m. until 11 p.m.; 3-h interval).

### 2.2 Chemicals and materials

A commercial standard of sirolimus (rapamycin) was acquired from Supelco (Pennsylvania, USA). Sirolimus-d3 solution was obtained from Sigma (Massachusetts, USA) and used as an internal standard (IS). For the Ultra High-Performance Liquid Chromatography (UHPLC) analyses, formic acid, p. a., ACS reagent (Carlo Erba, Milano, Italy), methanol (MeOH) ≥ 99,9% LC-MS reagent grade (VWR, Pennsylvania, USA), and ammonium formate for LC-MS, ≥99.0% (Supelco®, Pennsylvania, USA) were used. The supported liquid extraction (SLE) of sirolimus from breast milk was performed on Novum SLE MAX 96-Well Plate (Phenomenex, California, USA). Ethyl acetate ≥99,7% was from Honeywell (New Jersey, USA), zinc sulphate heptahydrate used for protein precipitation was from Sigma-Aldrich (Massachusetts, USA). Finally, before UHPLC tandem mass spectrometry (UHPLC-MS/MS) analysis, all extracts were filtered through 0.20 μm PTFE syringe filters (Chromafil® Macherey-Nagel, Germany).

### 2.3 Sirolimus quantitation in blood

The patient with TSC was on sirolimus therapy (4 mg/day) at steady state, with dose adjustments guided by therapeutic drug monitoring. On the 10th day postpartum, breast milk samples were collected. During the 24 h that breast milk was produced, the patient took sirolimus at 11 a.m. on both days (at the start and ending of breast milk collection period). On the 10th day postpartum, maternal whole blood concentrations of sirolimus were 5.9 ng/mL (11 a.m.) at the beginning of breast milk collection and immediately before sirolimus dose on that day. Elecsys® Sirolimus immunoassay was used for the *in vitro* quantitative determination of sirolimus in the patient’s whole blood.

### 2.4 Sirolimus quantitation in breast milk

#### 2.4.1 Extraction of sirolimus from breast milk samples

In order to optimize the extraction of sirolimus from breast milk, blank samples (breast milk from healthy women) were spiked with a sirolimus pure standard, to obtain a final concentration of 1 ng.mL^-1^ in milk. The extraction of the drug in plasma has usually been carried out using a protein precipitation method with acetonitrile (ACN), methanol (MeOH) and zinc sulfate (ZnSO_4_). (Polson et al., 2003; Pablo et al., 2020; Nguyen et al., 2021). Therefore, as a first approach, a protein precipitation was performed, testing different combinations of these solvents. However, probably due to the milk’s high protein content, these experiments did not allow the detection of 1 ng.mL^-1^ in milk. Therefore, in order to improve the extraction of sirolimus from breast milk, a supported-liquid extraction (SLE) method was optimized, based on a procedure previously described. (Shigeta et al., 2020). The optimized conditions involved the addition of 70 μL of ZnSO_4_ to 100 μL of milk sample, followed by the addition of 330 μL of sirolimus-d3 at 2 ng.mL^-1^ (IS) in cold ACN, for protein precipitation. After vortexing, samples were sonicated for 5 min and centrifuged at 13,500 rpm for 5 min.

The supernatant (400 μL) was then added to the microplate wells. After applying vacuum for 5 s and a 5-min incubation, 500 μL of ethyl acetate was added. After 5 min, vacuum was applied for 30 s. This extraction was performed three times. The ethyl acetate extracts were then evaporated until dryness, in a water bath at 40 °C in a fume hood, overnight. After evaporation, samples were reconstituted in 100 μL of MeOH and filtered immediately before analysis. All samples were prepared in triplicate.

#### 2.4.2 UHPLC–MS/MS analysis

Sirolimus quantitation in breast milk was performed by ultra-high performance liquid chromatography tandem mass spectrometry (UHPLC–MS/MS) using an Acquity™ Arc™ System (Waters®, Ireland) equipped with a quaternary pump, solvent degasser, autosampler, and column oven. The mass spectrometer used was a Xevo™ TQ-S cronos triple quadrupole (Waters®, Ireland). MassLynx® software (version 4.2) was used to control the equipment and for data acquisition and treatment.

The separation was performed in a Purospher STAR C18 column (50 × 2.1) mm, 2 μm, Supelco® (Darmstadt, Germany), kept at 50 °C, using 5 mM ammonium formate with 0.1% HCOOH in water (eluent A) and MeOH (eluent B) as eluents, with a total run time of 8 min at a flow rate of 0.3 mL min^-1^. The gradient of eluents was programmed from 60% eluent B to 85% over 0.5 min, followed by an increase to 95% eluent B over 1.5 min, held at 95% B for 2.9 min, and then returned to initial conditions over 3 min.

The ionization of the compounds was performed using an electrospray ionization source (ESI). Standard solutions of sirolimus and IS at 100 μg.mL^-1^, in methanol, were infused into the mass spectrometer in order to determine the two product ions with the highest signals. Sirolimus and IS exhibited ammonium adducts [M + NH_4_]^+^ at *m/z* 931.6 and 934.6, respectively, as previously described (Pablo et al., 2020; Nguyen et al., 2021; Gong et al., 2019; Treder et al., 2023). In the product ion spectra Supplementary Figure S1, the most prominent fragment ions were at *m/z* 864.6 for sirolimus and IS, which is in accordance with previous findings (Pablo et al., 2020; Nguyen et al., 2021; Gong et al., 2019; Treder et al., 2023). The capillary was set to 3.0 kV, source temperature to 150 °C, and desolvation temperature to 350 °C.

After chromatographic optimization, the analyses were performed in MRM (multiple reaction monitoring) mode in order to achieve higher selectivity and sensitivity. Two transitions were used for the analysis of sirolimus Supplementary Table S2. MRM 1 was used as the quantitation transition and MRM 2 as the confirmation transition, in order to evaluate the method specificity. High purity nitrogen (N_2_) was used both as drying gas and as a nebulizing gas. Ultra-high purity argon (Ar) was used as collision gas.

#### 2.4.3 UHPLC–MS/MS method validation

The performance and suitability of the method for the quantitation of sirolimus in breast milk were assessed by a series of validation experiments, including selectivity and carry-over, method sensitivity, linearity, accuracy, precision, matrix effect, and recovery.

Three breast milk samples (blank samples), from three different donors, were analyzed in triplicate for the evaluation of possible interferents (method selectivity). Carry-over was assessed by the analysis of solvent (MeOH) after nine consecutive injections of extracted blank samples at the upper limit of quantitation (160 pg. mL^-1^) and after nine consecutive injections of the IS (sirolimus-d3) at 0.528 ng.mL^-1^, the concentration corresponding to 100% recovery.

The limit of detection (LOD) and lower limit of quantitation (LLOQ) were determined in solvent and blank matrix (blank breast milk samples after extraction). In order to access accuracy and precision, three extracted milk blanks were spiked with sirolimus standard at the LLOQ (40 pg. mL^-1^), and mid (100 pg. mL^-1^) and high (160 pg. mL^-1^) concentrations. In order to evaluate the intra-day accuracy and precision, ten samples at each concentration level were injected into the equipment. Inter-day accuracy and precision were evaluated in three different days. Precision was also evaluated at the repeatability level (injection of the same sample 10 times).

Three calibration curves in solvent (MeOH), with eight calibration levels, from 40 to 160 pg. mL^-1^, were prepared from three independent solutions, to evaluate method linearity. The interference in the detector response due to the presence of unintended analytes or other interfering substances in the matrix (breast milk after extraction) – matrix effect (ME) was evaluated. To this aim, three calibration curves were prepared by spiking independent blank breast milk matrices with sirolimus, in order to obtain eight calibration levels, from 40 to 160 pg. mL^-1^. The slopes of these curves were compared with the slopes for pure solvent. The IS-normalized matrix factor (MF) was calculated by dividing the MF of the analyte by the MF of the IS, at the LLOQ (40 pg. mL^-1^), and medium (100 pg. mL^-1^) and high (160 pg. mL^-1^) concentrations, in duplicates.

The extraction efficiency of the analytical process (method recovery) was evaluated and reported as a percentage of a known amount of sirolimus carried through the sample extraction and processing steps of the method (U.S. Food and Drug Administration, Center for Drug Evaluation and Research, 2018; European Commission, 2002). The recovery of the analyte was optimized to ensure that the extraction was efficient and reproducible. (U.S. Food and Drug Administration, Center for Drug Evaluation and Research, 2018). To this aim, before extraction, three blank milk samples were spiked with 140, 200, and 300 pg. mL^-1^ of sirolimus, corresponding to theoretical concentrations (100% recovery) of 112, 160, and 240 pg. mL^-1^ in the final extract. Each sample was prepared in triplicate. For the recovery determination (%), three extracted blanks were spiked with these concentrations of sirolimus.

#### 2.4.4 Quantitation of sirolimus in breast milk samples

The validated method was applied to the quantitation of sirolimus in 18 breast milk samples collected from the TSC patient. Sirolimus was extracted and quantitated as previously stated.

## 3 Results

### 3.1 UHPLC–MS/MS method validation

The retention time of both sirolimus and IS was 4.10 ± 0.01 min. The method was selective for the analysis of sirolimus, since no peaks were detected in the blanks at the retention time of sirolimus and sirolimus-d3 (IS) standards. Similarly, no carry-over was detected for sirolimus nor IS. The limit of detection (LOD) of sirolimus in solvent (MeOH) was 10 pg. mL^-1^ Supplementary Figure S2, corresponding to a signal-to-noise of approximately 2.5:1, and the lower limit of quantitation (LLOQ) was 40 pg. mL^-1^, corresponding to a signal-to-noise of approximately 10:1 Supplementary Figure S2. The LOD and LLOQ in the extracted matrix were identical.

The method was linear from 40 to 160 pg. mL^-1^, with a coefficient of determination (R^2^) > 0.995, in solvent and matrix Supplementary Figure S3. The back calculated concentrations of the calibration standards were within 10%, surpassing the criteria described in different guidelines. (U.S. Food and Drug Administration, Center for Drug Evaluation and Research, 2018; Committee for Medicinal Products for Human Use CHMP, 2011). The method was considered precise and accurate for the quantitation of sirolimus. Their mean calculated concentrations were within ±9% of their nominal values Supplementary Table S3, lower than the established value of 15%. (U.S. Food and Drug Administration, Center for Drug Evaluation and Research, 2018; Committee for Medicinal Products for Human Use CHMP, 2011). Similar results were obtained for precision Supplementary Table S3.

The average ME of sirolimus was 85% ± 3% Supplementary Table S4 and the coefficient of variation (CV) of the IS-normalized MF Supplementary Table S5 was 9.0%, below the reference value of 15%. (Committee for Medicinal Products for Human Use CHMP, 2011). Thus, the addition of IS allowed an accurate quantitation of sirolimus in milk.

Recoveries of sirolimus varied between 18% to 38% Supplementary Table S6. Even though recoveries were below 50%, the method was able to detect 100 pg of sirolimus per mL of breast milk (LOD in milk) and to quantitate 140 pg. mL^-1^ (LLOQ in milk) Supplementary Figure S4 with suitable precision and accuracy Supplementary Table S7, which were in line with the most recent guidelines. (U.S. Food and Drug Administration, Center for Drug Evaluation and Research, 2018; Committee for Medicinal Products for Human Use CHMP, 2011).

### 3.2 Quantitation of sirolimus in breast milk samples

The evaluation of breast milk samples produced during 24 h following sirolimus administration was performed, to accurately define sirolimus secretion into maternal milk and possibly contribute to breast feeding guidance adjustment in the future.

Maternal sirolimus blood levels were found to be 5900 pg. mL^-1^ at the time of the first breast milk collection (11 a.m., 10th day postpartum). Infant blood concentrations of sirolimus were not assessed, as the patient had been advised not to breastfeed. Sirolimus was detected in all breast milk samples from the TSC patient, with concentrations ranging from 175 to 492 pg. mL^-1^ Table 1. It was possible to detect both MRM1 and MRM2 transitions in all samples, confirming the identification of the drug.

**TABLE 1:** Concentration of sirolimus in breast milk samples. Samples were collected on the 10th and 15th day postpartum.

Samples		Concentration (pg.mL^-1^)
10th day postpartum	11 a.m*	307 ± 9
	2 p.m.	243 ± 17
	5 p.m	245 ± 4
	8 p.m	313 ± 17
	11 p.m	173 ± 6
11th day postpartum	2 a.m	181 ± 2
	5 a.m	195 ± 13
	8 a.m	175 ± 7
	11 a.m	214 ± 7
15th day postpartum	2 a.m	262 ± 9
	5 a.m	248 ± 15
	8 a.m	280 ± 7
	11 a.m*	353 ± 19
	12 p.m	492 ± 21
	2 p.m	325 ± 5
	5 p.m	253 ± 2
	8 p.m	357 ± 9
	11 p.m	218 ± 13

* Sirolimus administration at 11 a.m.

## 4 Discussion

Pregnant patients medicated with sirolimus are generally patients with complex situations of solid organ transplant or TSC, and as such, have an increased risk of prematurity. (Li et al., 2019; Fiocchi et al., 2016; Kociszewska-Najman et al., 2017; Gomez-Casado et al., 2025). The benefits of breastfeeding have long been reported to improve child’s growth and development. The World Health Organization recommends exclusive breastfeeding for the first 6 months and continued complementary breastfeeding for at least 3 years. (WHO. Protecting, 2017). Benefits reported in the literature include reduced rates of infectious diseases and deaths as well as a decreased risk of future overweight/obesity and diabetes. (Sankar et al., 2015; Yan et al., 2014; Weng et al., 2012).

Particularly in preterm infants, breastfeeding seems to reduce the risk of conditions such as necrotizing enterocolitis and late-onset sepsis, bronchopulmonary dysplasia and retinopathy of prematurity and is associated with better neurodevelopmental results and enhanced cardiac performance. (Ip et al., 2009; Hylander et al., 1998; Schanler, 2001; Okamoto et al., 2007; El-Khuffash et al., 2021).

Considering the higher risk of preterm births in patients taking sirolimus for their underlying conditions, breastfeeding could be essential to decrease the risks associated with prematurity and improve infant short and long-term health. (Gomez-Casado et al., 2025). However, to date, pediatric guidelines discourage breastfeeding in women treated with sirolimus due to the lack of human data on sirolimus concentration in breast milk. (Li et al., 2019; E-lactation, 2025).

Considering sirolimus high molecular weight (914.2 g/mol) and plasma protein binding, along with low oral availability, its excretion into breast milk and absorption by the newborn seems unlikely (Mahalati and Kahan, 2001; E-lactation, 2025; Gomez-Casado et al., 2025). Therefore, to detect sirolimus concentrations in breast milk an UHPLC-MS/MS was developed and validated. Protein precipitation is essential for extraction because sirolimus extensively binds to proteins (Mahalati and Kahan, 2001) Thus, it was necessary to disrupt the protein–drug binding so that the total amount of sirolimus could be extracted for analysis. (Polson et al., 2003). Possibly due to their high protein content, the extraction of sirolimus from milk was challenging. In this way, after protein precipitation, a further SLE extraction method was developed and optimized based on a previously reported protocol. (Shigeta et al., 2020).

The optimization step was crucial, as sirolimus concentrations in breast milk samples ranged from 173 to 492 pg. mL^-1^. Maximum sirolimus levels in breast milk were observed one hour after oral administration, consistent with the typical time to peak concentrations (0.5–3 h post-dose) (Mahalati and Kahan, 2001) However, this conclusion is limited by the absence of corresponding serum sirolimus concentrations at this time point.

The developed method allowed the detection of 100 pg of sirolimus per mL of milk, and the quantitation of 140 pg. mL^-1^, a value markedly lower than previously reported limits of quantitation for sirolimus in blood (500–2000 pg. mL^-1^). (Pablo et al., 2020, Shigeta et al., 2020, Nguyen et al., 2021, Gong et al., 2019, Yatscoff, 1996).

In our case study, sirolimus was restarted during pregnancy to treat a life threatening fetal cardiac rhabdomyoma diagnosed in uterus, as both mother and fetus had TSC. However, as the newborn was stable, the pediatric team considered that sirolimus was not to be initiated and that breastfeeding would not be indicated, given the absence data about the safety of the milk concentrations of sirolimus that were found (173–492 pg. mL^-1^) for newborns. However, available evidence suggests that sirolimus is well tolerated even when used in therapeutic levels in the first year of life. (Cavazos et al., 2024). Several studies have reported sirolimus therapeutic use in newborns to treat vascular malformations and lymphatic disorder, most of whom had minor transient side effects and 27% had no adverse effects at all. (Cavazos et al., 2024). Furthermore, based on the information from the literature, the lowest therapeutic dose of sirolimus in a 1-month old with 0.26 m^2^ would be around 0.13 mg/day (Cavazos et al., 2024; Serio et al., 2022), corresponding to an estimated intake of at least 264 L of breast milk. By comparison, the infant’s average daily breast milk intake is approximately 0.624 L (Rios-Leyvraz and Yao, 2023) Thus, exposure of the newborn to sirolimus is expected to be much lower than its therapeutic dose. (Serio et al., 2022).

However, this study has several limitations that should be acknowledged. First, paired maternal blood concentrations of sirolimus were not available at all time points corresponding to breast milk sampling, which limits the ability to establish a direct relationship between plasma and milk concentrations. Additionally, although the detected concentrations in breast milk suggest low exposure, the clinical effects of such low levels of sirolimus in newborns remains insufficiently characterized. These limitations highlight the need for further studies to better define the pharmacokinetics and safety profile of sirolimus during breastfeeding. Clinicians providing guidance on breastfeeding during sirolimus treatment should consider monitoring infants for known adverse effects, despite the expected low exposure. If clinical concerns arise, therapeutic drug monitoring of the infant may be considered. Additionally, the reporting and publication of such clinical observations should be encouraged to enhance the evidence base.

## 5 Conclusion

Sirolimus is widely used in solid organ transplantation and increasingly in TSC manifestations. Using a validated UHPLC-MS/MS method, sirolimus concentrations in breast milk from a TSC patient were quantitated, ranging from 173 to 492 pg. mL^-1^, suggesting that infant exposure is likely far below therapeutic levels. Although more studies are needed to characterize infant serum sirolimus concentrations and confirm safety, these findings provide valuable evidence to inform discussions on breastfeeding in patients treated with sirolimus.

## Data Availability

The original contributions presented in the study are included in the article/Supplementary Material, further inquiries can be directed to the corresponding author.

## Supplementary material

Supplementary Figure S1 MS/MS spectra of sirolimus **(A)** and sirolimus-d3 **(B)**, applying a cone voltage of 30 V and collision energy of 14 eV.

Supplementary Figure S2 Chromatogram of the quantification transition (MRM 1: 931.6 > 864.6) of sirolimus at 10 pg.mL-1 **(A)**, corresponding to the limit of detection (LOD), and at 40 pg.mL-1 **(B)**, corresponding to the lowest limit of quantitation (LLOQ).

Supplementary Figure S3 Calibration curves of sirolimus in MeOH and matrix.

Supplementary Figure S4 Chromatogram corresponding to the quantification transition (MRM 1: 931.6 > 864.6) of sirolimus at 100 pg.mL^-1^, corresponding to the limit of detection (LOD) in milk **(A)** and at 140 pg.mL^-1^, corresponding to the lowest limit of quantification (LLOQ) in milk **(B)**.

Supplementary Table S1 ClinPK checklist. n/a: not applicable.

Supplementary Table S2 Multiple reaction monitoring (MRM) conditions used for the quantification of Sirolimus and sirolimus-d3 (internal standard). CV: Cone Voltage; CE: Collision Energy.

Supplementary Table S3 Intra-day and inter-day precision and accuracy of sirolimus in blank samples. Results are expressed as %.

Supplementary Table S4 Matrix effect on sirolimus determination in different blank samples. MF: Matrix Factor.

Supplementary Table S5 Internal standard (IS)-normalized matrix factor (MF) of sirolimus in different blank samples, at three different concentration levels in the matrix.

Supplementary Table S6 Method recovery of sirolimus extraction in different blank samples, at three calibration levels in milk. Results are expressed as %.

Supplementary Table S7 Precision and accuracy of sirolimus extraction in different blank samples, at three calibration levels in milk. Results are expressed as %.
