# Supplementary material for: Sirolimus excretion in breast milk: a preliminary investigation
Source: Front Pharmacol. 2026 Jun 26;17:1859156. doi: 10.3389/fphar.2026.1859156 (PMC13350175; doi:10.3389/fphar.2026.1859156)
Supplement: Supplementary file 1 [file DataSheet1.pdf]

Supplementary Material

1 Supplementary Figures and Tables

1.1 Supplementary Figures

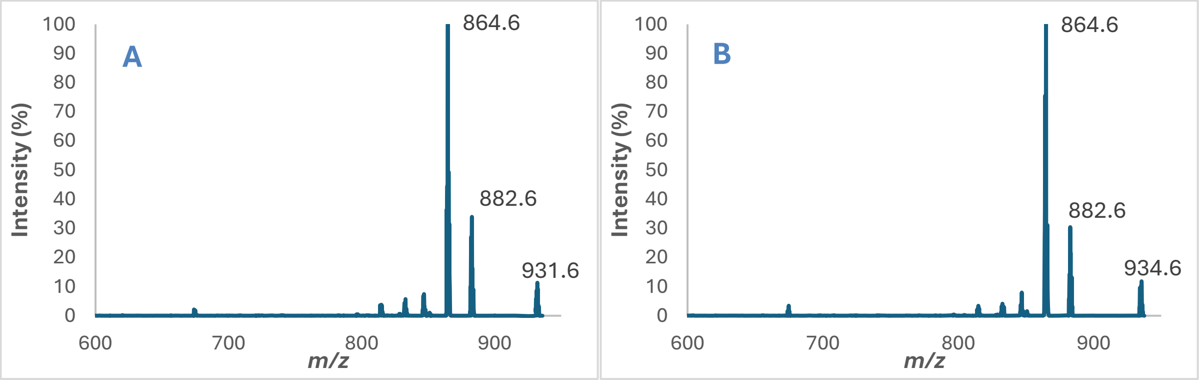

Supplementary Figure 1. MS/MS spectra of sirolimus (A) and sirolimus-d3 (B), applying a cone voltage of 30 V and collision energy of 14 eV.

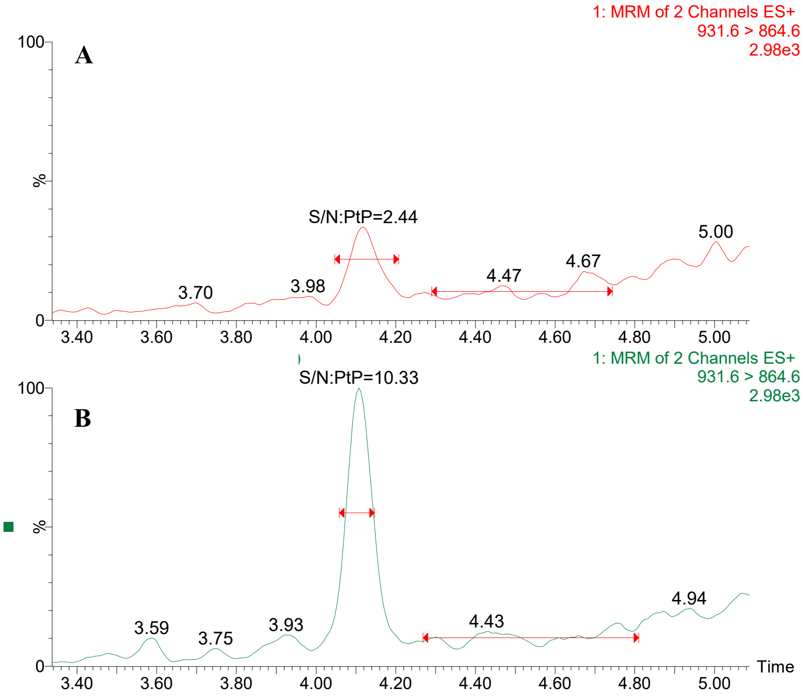

Supplementary Figure 2. Chromatogram of the quantification transition (MRM 1: 931.6 > 864.6) of sirolimus at 10 pg.mL^{-1} (A), corresponding to the limit of detection (LOD), and at 40 pg.mL^{-1} (B), corresponding to the lowest limit of quantification (LLOQ).

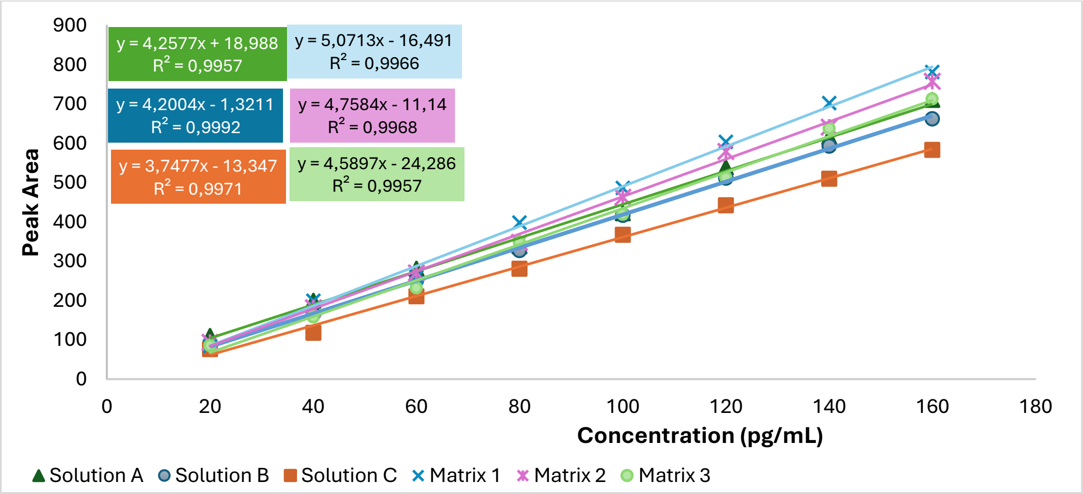

Supplementary Figure 3. Calibration curves of sirolimus in MeOH and matrix.

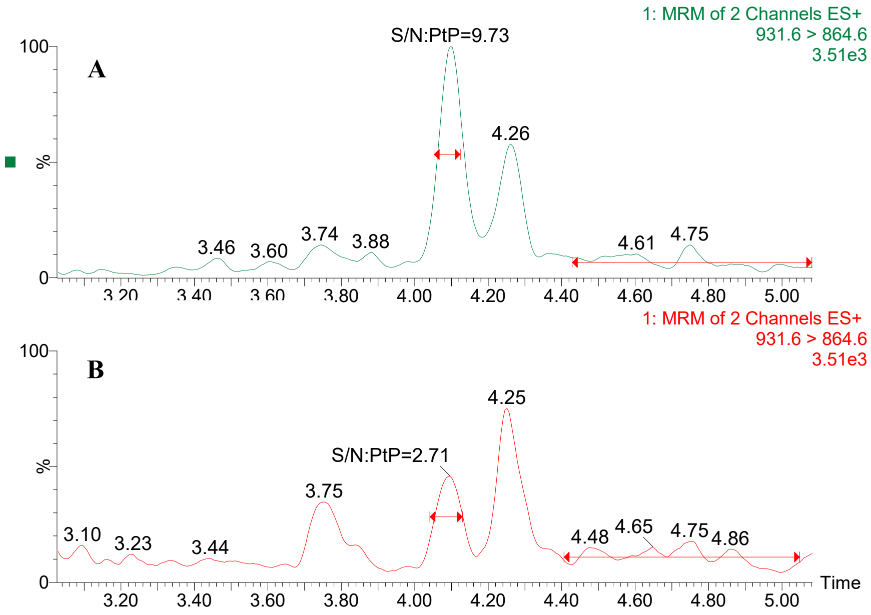

Supplementary Figure 4. Chromatogram corresponding to the quantification transition (MRM 1: 931.6 > 864.6) of sirolimus at 100 pg.mL^{-1}, corresponding to the limit of detection (LOD) in milk (**A**) and at 140 pg.mL^{-1}, corresponding to the lowest limit of quantification (LLOQ) in milk (**B**).

1.2 Supplementary Tables

Supplementary Table 2. Multiple reaction monitoring (MRM) conditions used for the quantification of Sirolimus and sirolimus-d3 (internal standard).
CV: Cone Voltage; **CE:** Collision Energy.

Compound	Precursor ion (*m/z*)	CV (V)	MRM1	CE (eV)	MRM2	CE (eV)
Sirolimus	931.6	30	864.6	14	882.6	10
Sirolimus-d3	934.6	30	864.6	14	882.6	10

Supplementary Table 2. Intra-day and inter-day precision and accuracy of sirolimus in methanol. Results are expressed as %.

Concentration ($pg.mL^{-1}$)	Intra-day precision		Inter-Day Precision	Intra-Day Accuracy	Inter-Day Accuracy
	Injection Repeatability	Experimental Repeatability			
**40**	5.7	5.2	5.7	4.8	6.0
**100**	6.4	5.9	5.7	1.4	5.6
**160**	1.8	7.3	6.8	2.8	7.0

Supplementary Table 3. Matrix effect on sirolimus determination in different blank samples.
MF: Matrix Factor.

	Solvent	Blank 1	Blank 2	Blank 3	Average (matrix)
**Slope**	4.12 ± 0.24	5.07	4.83	4.72	4.87
**MF (%)**	100	81	85	87	85 ± 3.0

Supplementary Table 4. Internal standard (IS)-normalized matrix factor (MF) of sirolimus in different blank samples, at three different concentration levels in the matrix.

Concentration ($pg.mL^{-1}$)	Blank 1	Blank 2	Blank 3
**40**	96 ± 9.2	86 ± 16.5	89 ± 9.4
**100**	93 ± 2.2	99 ± 0.4	99 ± 9.6
**160**	100 ± 8.2	94 ± 1.6	104 ± 11.3

Supplementary Table 5. Method recovery of sirolimus extraction in different blank samples, at three calibration levels in milk. Results are expressed as %.

Concentration ($pg.mL^{-1}$)	Blank 1	Blank 2	Blank 3
**140**	18 ± 4.1	20 ± 0.5	38 ± 4.8
**200**	22 ± 1.4	21 ± 3.1	30 ± 0.9
**300**	25 ± 12.1	24 ± 1.9	33 ± 8.3

Supplementary Table 6. Precision and accuracy of sirolimus extraction in different blank samples, at three calibration levels in milk. Results are expressed as %.

Concentration ($pg.mL^{-1}$)	Blank 1		Blank 2		Blank 3	
	Precision	Accuracy	Precision	Accuracy	Precision	Accuracy
**140**	6.8	0.3	2.0	5.3	6.0	12.6
**200**	7.9	0.7	7.0	6.8	6.1	2.5
**300**	6.7	2.2	5.1	0.4	1.8	3.6
